# Making the best of the worst: Care quality during emergency cesarean sections

**DOI:** 10.1371/journal.pone.0227988

**Published:** 2020-02-21

**Authors:** Betina Ristorp Andersen, Maria Birkvad Rasmussen, Karl Bang Christensen, Kirsten G. Engel, Charlotte Ringsted, Ellen Løkkegaard, Martin G. Tolsgaard

**Affiliations:** 1 Department of Gynaecology and Obstetrics, Hillerød Hospital, Northzealand Hospital, University of Copenhagen, Hillerød, Denmark; 2 Copenhagen Academy of Medical Education and Simulation, Rigshospitalet, Capital Region of Denmark, Copenhagen, Denmark; 3 Department of Gynaecology and Obstetrics, Zealand University Hospital, Roskilde, Denmark; 4 Department of Health, Section of Biostatistics, University of Copenhagen, Øster Farimagsgade, Copenhagen, Denmark; 5 Center for Health Science Education, Faculty of Health, Aarhus University, Aarhus, Denmark; 6 Department of Obstetrics, Rigshospitalet, University of Copenhagen, Copenhagen, Denmark; Chinese Academy of Medical Sciences and Peking Union Medical College, CHINA

## Abstract

**Objective:**

This study aimed to identify factors influencing mothers’ and their partners’ perceptions of care quality, and to identify associated clinical factors.

**Methods:**

Questionnaires were developed based on eight interviews with couples after emergency Cesarean Sections (ECS). The internal structure of the questionnaires was examined using Rasch analysis. Cronbach’s alpha was calculated to evaluate internal consistency of questionnaire items. Finally, associations between questionnaire scores and ECS characteristics were determined.

**Results:**

Thematic analysis of interview data demonstrated that team-dynamics, professionalism, information, safety, leadership and mother-child continuity of care are important to patient- perceived quality of care. Questionnaire responses from 119 women and 95 partners were included in the validation and demonstrated satisfying fit to the Rasch model. The questionnaires had acceptable internal consistency with Cronbach’s alpha 0.8 and 0.7 for mothers and partners, respectively. Perceived quality of care was negatively associated with increasing urgency of the CS. Spearman rank correlation coefficients were -0.34 (p <0.001) and -0.32 (p = 0.004) for mothers and partners, respectively. Perceived quality of care differed significantly across CS indications for both mothers (p = 0.0006) and their partners (p<0.0001).

**Conclusion:**

Team-dynamics, professionalism, information, safety, leadership and mother-child-continuity affect patients’ perceptions of care. Perceptions of care were highly influenced by CS indications and urgency.

## Introduction

Patient-perceived quality of care during childbirth may be associated with considerable psychologic morbidity and affect maternal-infant bonding [1,2]. In obstetric care, maternal satisfaction has been identified as an integral part of care quality [3–6]. Accordingly, patient-perceived quality of care has received increased attention in North America and Europe over the past decade [7–10]

Studies on labor experiences for women undergoing vaginal births have found associations between patient-perceived quality of care and caregivers’ non-technical skills, as well as numerous maternal factors, including education levels, levels of anxiety, mode of delivery and pain [3,4,11–13]. Studies have demonstrated higher maternal satisfaction after spontaneous vaginal deliveries compared with emergency cesarean sections (ECS) [1,14–16]. Although ECS are associated with the lowest satisfaction during childbirth [2], little is known about which factors contribute to mothers’ and their partners’ perception of care quality in situations where an ECS is required. The indication for a cesarean section (CS) may influence mothers’ and partners’ perception of quality of care, as CS performed due to arrested labor may be associated with lower patient satisfaction compared with CS performed due to perceived risk to the mother or child [17]. However, such correlations may also be the result of decreased information delivery to mothers and their partners as the level of urgency of the CS increases [18]. To our knowledge, there are no prior studies which compare the urgency of CS and mothers’ and their partners’ perception of care quality. It is likely that some of the factors that result in poor maternal satisfaction during ECS can be modified if they are identified and addressed during the training of midwives, obstetricians, and anesthetists.

Hence, the objectives of this study were 1) to identify factors influencing mothers’ and their partners’ perceptions of care quality in the context of ECS; 2) to develop questionnaires and examine their validity in a large group of women and partners; and finally, 3) to explore how urgency classification and CS indications are associated with mothers’ and their partners’ perceived quality of care.

## Material and methods

**Fig 1:**
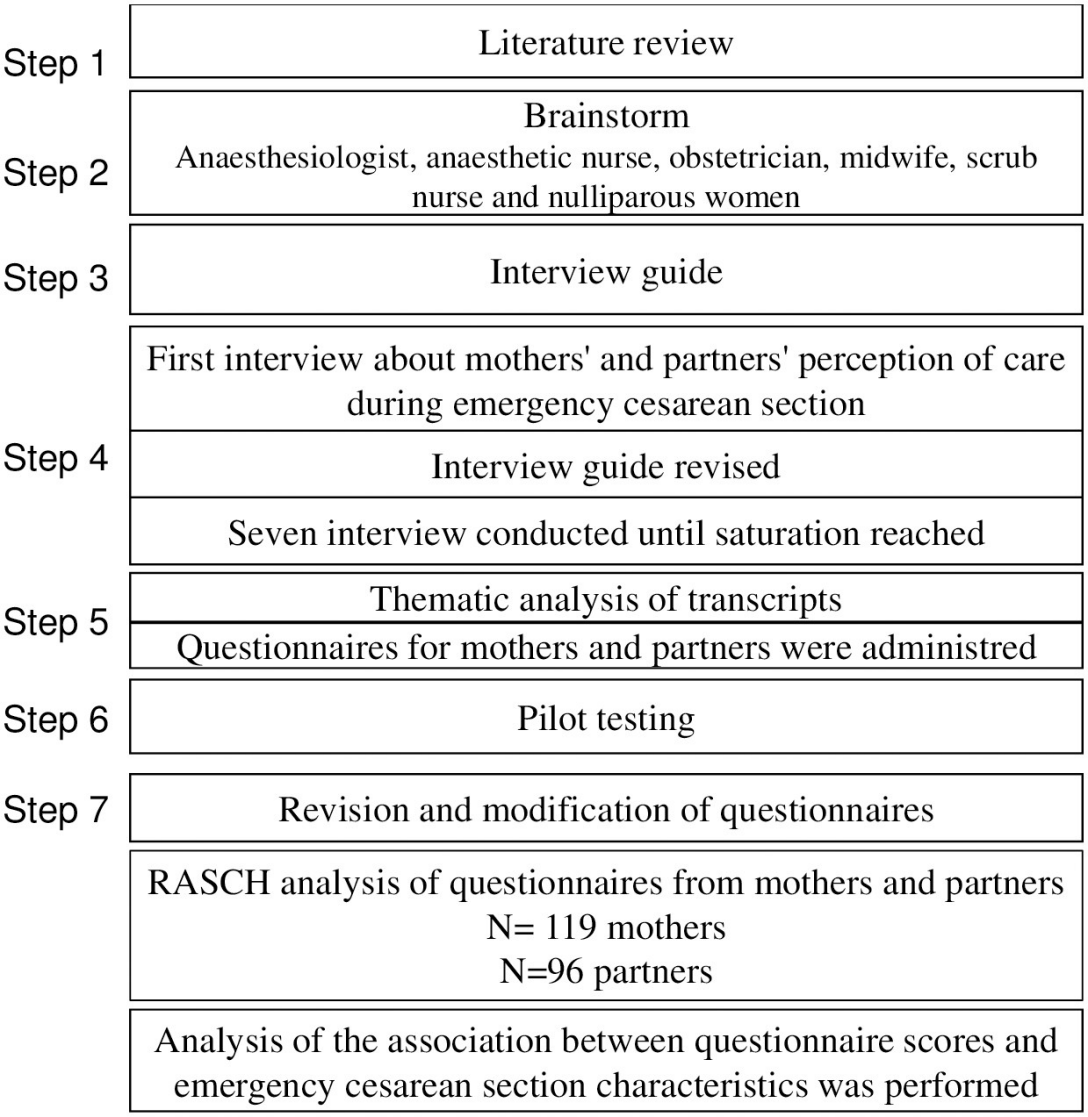
Study process and questionnaire development.

In this study, we defined quality of care as the elements which matters to *patients and their partners*, aiming to understand which factors influenced their perceptions of quality of care and how to measure it [19]. The study was conducted at Hillerod Hospital in Denmark between June 2015 and October 2017. The study was conducted in three parts: first, a literature review [1,2,4,11,12,15,17,20–32] and brainstorm among a multi-professional team involved in ECS resulted in a semi structured interview guide (S1 Appendix). Subsequently, in-depth interviews with mothers and their partners were performed. Factors that influenced the perceived quality of care from the transition to ECS until arrival on the maternity ward were identified. In step two, questionnaires were developed based on the interview data. A Rasch analysis of the internal structure based on responses from a large group of women and their partners was refined. Finally, in step three, we examined the association between questionnaire scores and ECS characteristics, including urgency classification and CS indications (Fig 1).

The study was approved by the Danish Patient Safety Authority (3-3013-1732/1). Data was reported to the Danish data protection agency (NOH-2015-040. I-Suite: 04287). Informed written consent was obtained from all participants.

### Step 1: Interviews

A stepwise approach was used to develop the interview guide which, in turn, was utilized to obtain information from mothers and their partners [33]. First, a literature review was performed to identify key questions relating to quality of care during ECS. Second, a multidisciplinary group, including anesthesiologists, obstetricians, anesthetic nurses and scrub nurses, midwifes, and nulliparous women, discussed the key themes identified in the literature review and gave further input regarding a list of questions that could provide information about care quality from mothers and their partners. BRA led the discussion, first presenting the results of the literature review and then inviting suggestions for questions to be included in the interview guide. All generated questions were included in the subsequent interview guide for mothers and partners.

Mothers, who underwent ECS from June to August 2015, and their partners were eligible for interview. The first author (BRA) conducted face-to-face interviews with the participants on the maternity ward one to four days after the ECS. The interviews followed the semi-structured interview guide and lasted fifteen to thirty-five minutes.

The participating couples were interviewed together. Additional interviews were conducted until saturation was achieved. Data was analyzed for thematic content [34–38]. We accomplished this by coding data, discussing emerging themes and revising these themes in an iterative manner until agreement was achieved within the author group. During this process, we checked for coherence within themes and avoidance of overlap, despite the fact that some themes were related, i.e. ‘team dynamics’ and ‘professionalism’. BRA and the project midwife analyzed the data transcripts with an iterative approach until agreement was reached prominent themes (Fig 1).

### Step 2: Development and validation of questionnaires

**Fig 2:**
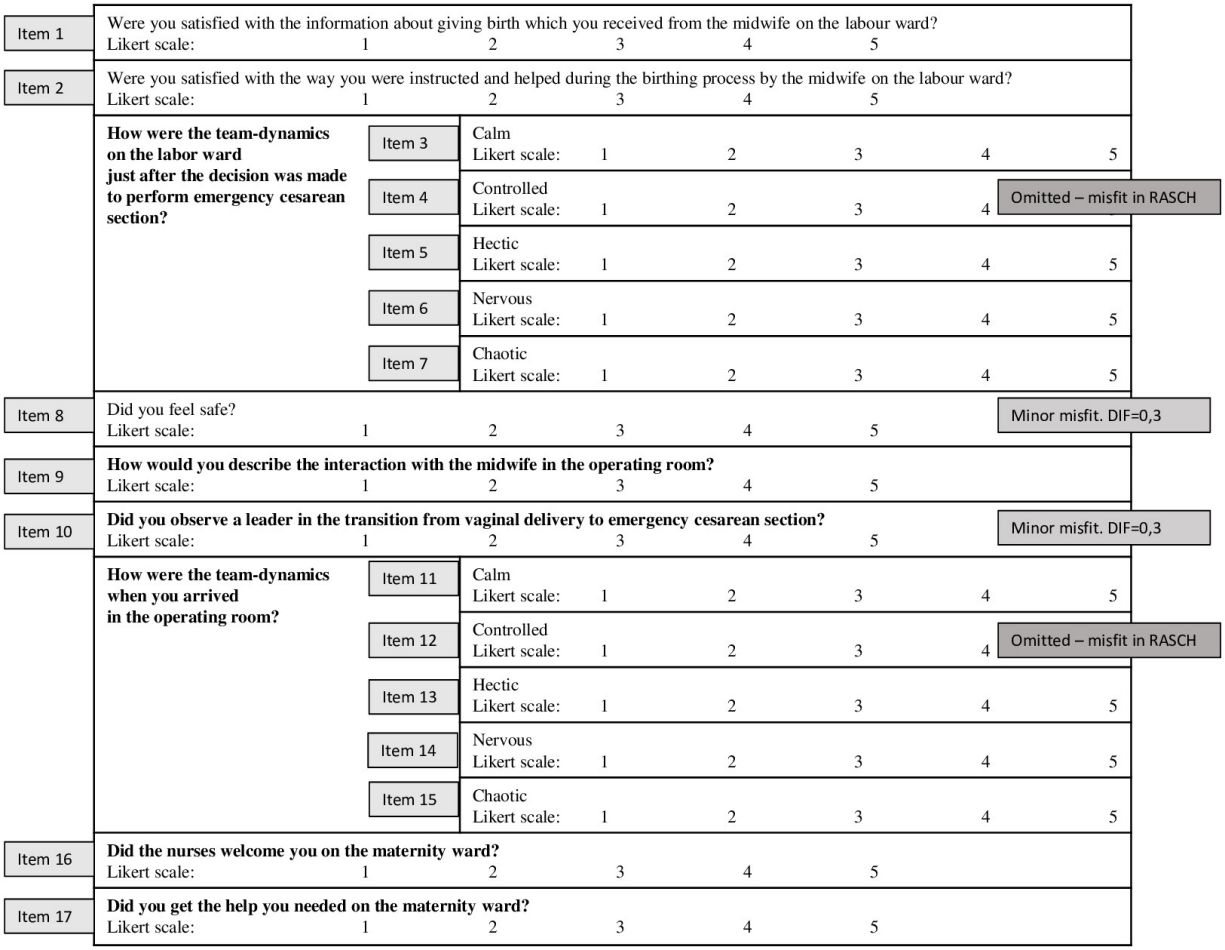
Maternal questionnaire. Likert scale is a visual analog scale that measure subjective characteristics. The Likert items state the level of disagreement = 1; agreement = 5. Misfit: Rasch analysis is a statistical methodology that tests if the observed item scores are consistent with measurement requirements. If this is not the case, there is a misfit. If the misfit is high the questionnaire item is omitted (left out).

**Fig 3:**
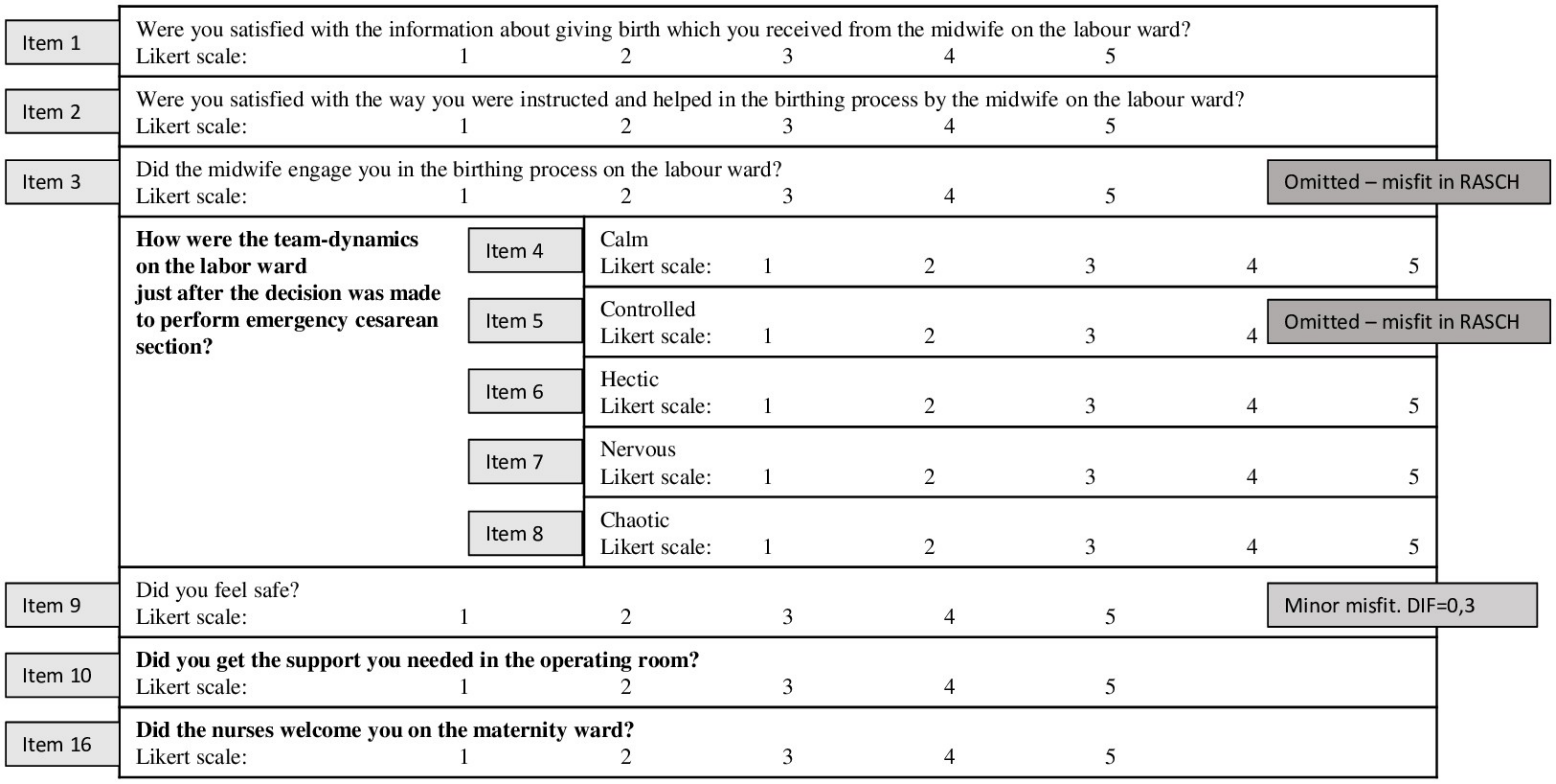
Partners´ questionnaire. Likert scale is a visual analog scale that measures subjective characteristics. The Likert items state the level of disagreement = 1; agreement = 5. Misfit: Rasch analysis is a statistical methodology that tests if the observed item scores are consistent with measurement requirements. If this is not the case, there is a misfit. If the misfit is high, the questionnaire item is omitted (left out).

A questionnaire was developed based on the themes obtained from the interviews with mothers and their partners (Figs 2 and 3). Cognitive pre-testing of the questionnaire was conducted during a pilot test with a small sample of mothers and their partners (n = 5). Pilot testing resulted in rewording of two questions. Subsequently, the questionnaires were distributed to a sample of 119 women and 95 partners.

#### Study participants

Mothers and partners who experienced ECS from January to October 2017 were eligible for inclusion. The mothers and partners answered the surveys on day 1–4 after the ECS. Clinical data was collected, including classification of urgency and indication for the CS. The classification of urgency was: 1: immediate threat to life of woman or fetus; 2: no immediate threat to life of woman or fetus but risk of adverse outcome with continued vaginal delivery and, therefore, transition to CS as soon as possible 3: No immediate danger to mother or fetus, but delivery by CS required for example due to arrested labor [39].

The indication for the CS was classified as either 1: suspected fetal distress, 2: labor dystocia 3: breech presentation 4: other.

#### Statistical methods

Internal consistency of the questionnaire was explored by calculating Cronbach’s alpha coefficients measuring the inter-item correlation. Alpha values above 0.7 were considered acceptable.

The questionnaires were validated by evaluating the fit of the data to an item response theory model [40], the Rasch model [41]. The Rasch model is a simple item response theory that has desirable statistical properties for validity testing. The Rasch model expresses the ideal measurement requirements, such that the individual questions in the questionnaire measure only one unidimensional variable and are both monotonous and locally independent. We evaluated overall fit of the model using the Andersen [42] conditional likelihood ratio test and individual item fit using Item-Rest score Association [43]. Local dependence was tested using the Q3 [44] statistic and differential item functioning (DIF) [45] for the two variables “urgency classification” and “indication” using log linear Rasch model tests [46].

Thus, the tests of differential item functioning ensured that the same construct was measured across indication subgroups ('suspected fetal distress', 'labor dystocia', 'breech presentation' and other), as well as across the subgroups defined by the urgency classification. We aimed to ensure that items were equally easy for respondents in different subgroups to endorse, if they had the same overall level of perceived quality of care. In educational research, the term 'test equating' refers to the statistical process of determining comparable scores on different forms of an exam [47]. Methods for doing this can be used to study the impact of DIF.

In all analyses, we controlled the false discovery rate (FDR) [48]. The false discovery rate is a method to conceptualize the rate of type I errors.

Beyond statistical tests, we evaluated validity graphically by plotting the observed item means (for each question) against the observed rest scores (total score minus the score for each corresponding question) and comparing these to 95% confidence intervals. To estimate the impact of differential item functioning, we plotted the equated scores to evaluate the magnitude.

### Step 3: Clinical factors associated with patient-perceived quality of care

The association between urgency classification (1,2,3) and the total score for patient-perceived quality of care measured by the questionnaire was evaluated using Spearman Rank correlations and partial Spearman rank correlations across indication strata.

In order to compare groups and understand the nature of disclosed group difference, the association between indication for CS and the total score of patient-perceived quality of care was evaluated using a Kruskal-Wallis test. Furthermore, Friedman’s two-way non-parametric ANOVA was performed to control for classification of urgency. In the latter analysis, pairwise comparison of subgroups using Tukey’s adjustment for multiple testing was used to find the indication subgroup with highest patient perceived quality of care.

## Results

### Step 1: Interviews

In total, ten couples received invitations to participate. Description of the population under study are shown I Table 1. Most of the women participating were primiparous and had grade 2 CS. The indications for the CS varied with half due to suspected asphyxia and the other half due to failed vacuum extraction, dystocia, and breech presentation. Two couples declined to participate due to fatigue. Saturation was reached after conducting the first six interviews. Two additional interviews were held, but no new information emerged.

**Table 1:** Description of study population from interviews.

Name	Age	GA	Parity	Prior CS	Induction of labor	Duration of labor	Emergency grade CS	Date of birth	Indication for CS
No 1	31	40+3	0	No	No	8 hours and 33 minutes	1	26.05.15	Failed delivery by vacuum
No 2	34	40+0	1	Yes	Braking the water	14 hours and 45 minutes	2	02.06.15	Dystocia of labor
No 3	33	39+6	0	No	No	Not started	2	07.07.15	CTG with deceleration prior to contractions
No 4	30	40+1	0	No	No	3 hours and 36 minutes	2	11.07.15	Asphyxia scalp-ph 7,13
No 5	32	39+5	0	No	No	4 hours and 38 minutes	2	12.07.15	Failed delivery by vacuum
No 6	41	41+2	0	No	Yes	13 hours and 56 minutes	2	11.07.11	Asphyxia Fetal tachycardia on cardiotocography
No 7	31	38+1	0	No	No	22 hours and 28 minutes	2	17.07.15	Failed delivery by vacuum
No 8	37	41+0	1	Yes	No	30 minutes	1	20.07.15	Leading part of baby was a knee

The following themes were identified: Team-dynamics, professionalism, information provided by team members, leadership and mother-child continuity of care (Table 2).

**Table 2:** Thematic analysis of interview transcripts with quotes.

Theme	Quote	Analysis
Team-dynamics	*“Suddenly there were 10 persons in the room [the labour ward] talking about cutting my clothes up (to speed things up)–I think I just blacked out*.*”* Another mother explained: “I*t was like a big chaos*, *the staff running around not knowing what other team members were supposed to do”*	The mothers and partners associated the feeling of control, respectful professional conversation and efficient task completion with a good team atmosphere. On the contrary, if the atmosphere became too hectic, the perception of quality decreased.
Professionalism	*“I was impressed by the way things happened*. *I felt a calm and professional atmosphere*. *I saw good communication between 10 professionals and everyone knew their tasks”*	The mothers and their partners commented on professionalism in terms of individual as well as team-performance. The participants emphasized a feeling of confidence when all team members knew their tasks and coordinated efficiently in a very intense emergency. Knowing the mothers’ history was also recognised as professionalism, while frustration occurred in case of change of midwifes resulted in additional examinations and repeated questions.
Information provided by team members	*“I heard the suction of blood*, *and I heard them measuring—I could hear it was a lot*, *and I thought*, *am I going to die*? *It creates the feeling of being unsafe”*.	We found contradictory results with regard to the need of information. In the transmission to operating room, some of the mothers and partners reported that they could not absorb any information. Other couples preferred description of all procedural steps in a clear language. After delivery and while still in the operating room, information was desired
Leadership	*”Being in labour for 42 hours*, *you start thinking*: *Is someone going to make a decision*?*”*	The need for leadership was considered important. The feeling of someone taking decisions and directing next actions was appreciated and gave a sense that the situation was under control.
Continuity of mother-child contact	*“It took 18 hours before I saw my son*. *What happened with him*? *Was he all alone*?*”*	After delivery, the opportunity to see and interact with the newborn was critical to the mothers. If the baby needed neonatal care, the mother and her child would be separated during the rest of the operation and this separation negatively influenced the overall perception of the CS for both the mother and her partner.

### Step 2: Validation of questionnaires

#### Questionnaires for mothers

The questionnaire consisted of items about information provided by team members, working environment (labor ward / operating room), leadership, and care on the maternity ward (Fig 3). A total of 119 women, who underwent an ECS, returned the questionnaires. There were very few missing data for the 119 mothers (3.2%). The maximum number of missing values for a single item was six. The answers from mothers are shown in Table 3.

**Table 3:** Distribution of item responses from 119 mothers. Legend: 1: Low perception of care, 5: High perception of care.

Item	1	2	3	4	5	Missing
Information about giving birth, which you received from the midwife on the labor ward?	0 (0%)	1 (1%)	5 (4%)	40 (34%)	73 (61%)	0 (0%)
Instruction and help during the birthing process from the midwife on the labor ward?	0 (0%)	1 (1%)	1 (1%)	17 (14%)	99 (83%)	1 (1%)
Team-dynamics at labor ward Calm?	13 (11%)	2 (2%)	9 (8%)	10 (8%)	80 (67%)	5 (4%)
Team-dynamics on labor ward Controlled?	15 (13%)	1 (1%)	2 (2%)	5 (4%)	91 (76%)	5 (4%)
Team-dynamics on labor ward Hectic?	15 (13%)	8 (7%)	6 (5%)	6 (5%)	79 (66%)	5 (4%)
Team-dynamics on labor ward Nervous?	2 (2%)	2 (2%)	3 (2%)	4 (3%)	103 (87%)	5 (4%)
Team-dynamics on labor ward Chaotic?	2 (2%)	3 (2%)	1 (1%)	5 (4%)	103 (87%)	5 (4%)
Did you feel safe?	4 (3%)	2 (2%)	4 (3%)	35 (30%)	74 (62%)	0 (0%)
Interaction with midwife in the operating room	0 (0%)	0 (0%)	22 (18%)	47 (40%)	46 (39%)	4 (3%)
Did you observe a leader during the procedure?	1 (1%)	2 (2%)	42 (35%)	28 (24%)	42 (35%)	4 (3%)
Team-dynamics—operating room Calm?	10 (8%)	2 (2%)	11 (9%)	15 (13%)	76 (64%)	5 (4%)
Team-dynamics—operating room Controlled?	11 (9%)	1 (1%)	1 (1%)	8 (7%)	93 (78%)	5 (4%)
Team-dynamics—operating room Hectic?	14 (12%)	9 (8%)	2 (2%)	17 (14%)	72 (60%)	5 (4%)
Team-dynamics—operating room Nervous?	1 (1%)	1 (1%)	3 (2%)	6 (5%)	102 (86%)	6 (5%)
Team-dynamics—operating room Chaotic?	3 (2%)	1 (1%)	2 (2%)	6 (5%)	102 (86%)	5 (4%)
Did you feel welcome on the maternity ward?	0 (0%)	0 (0%)	4 (3%)	21 (18%)	92 (77%)	2 (2%)
Did you get the help you needed on the maternity ward?	0 (0%)	2 (2%)	0 (0%)	22 (19%)	92 (77%)	3 (2%)

Initially, the questionnaire consisted of 17 questions. After the Rasch analyses, two questions pertaining to *the feeling of control in the delivery room* and *the feeling of control in the operating theater* were omitted from further analyses because of misfit. For the remaining 15 questions we found a good fit to the Rasch model (Andersen z = 60.1 df = 49, p = 0.13) (S2 Appendix).

After adjustment for multiple testing, no questions indicated misfit. Question 10 and question 16 had differential item functioning with respect to urgency of CS (S2 Appendix. Additional graphical analyses revealed all questions to be monotonous (mean values increasing with the total score) and disclosed minor misfit for question 8. The resulting sum score (with possible values ranging from zero to 60) had a mean of 52.3 and median 55 (IQR: 49 to 58). The impact of differential item functioning (S2 Appendix) was estimated by the equated scores. For question 10 and 16, the equated scores indicated that differential item functioning substantially inflated the total score in the group “urgency classification 3, requires early delivery” [34].

The reliability of the scale was good (Cronbach’s alpha 0.83).

#### Questionnaires for partners

In total, 95 partners returned questionnaires. There was very little missing data (1.5%). The maximum number of missing values for a single item was three. The answers from partners are shown in Table 4.

**Table 4:** Distribution of item responses from 95 partners. Legend: 1: Low perception of care, 5: High perception of care.

Item	1	2	3	4	5	Missing
Satisfaction with information form midwife on the labor ward?	0 (0%)	0 (0%)	7 (7%)	33 (35%)	55 (58%)	0 (0%)
Satisfaction with the instruction and help from midwife on the labor ward?	0 (0%)	0 (0%)	3 (3%)	20 (21%)	72 (76%)	0 (0%)
Did the midwife engage you in the birth process	7 (7%)	87 (92%)	0 (0%)	0 (0%)	0 (0%)	1 (1%)
Team-dynamics on labor ward Calm?	8 (8%)	3 (3%)	10 (11%)	11 (12%)	61 (64%)	2 (2%)
Team-dynamics on labor ward Controlled?	13 (14%)	1 (1%)	0 (0%)	12 (13%)	67 (70%)	2 (2%)
Team-dynamics on labor ward Hectic?	7 (7%)	6 (7%)	8 (8%)	8 (8%)	64 (68%)	2 (2%)
Team-dynamics on labor ward Nervous?	0 (0%)	3 (3%)	3 (3%)	5 (5%)	82 (87%)	2 (2%)
Team-dynamics on labor ward Chaotic?	1 (1%)	1 (1%)	1 (1%)	8 (8%)	82 (87%)	2 (2%)
Did you feel safe?	0 (0%)	0 (0%)	3 (3%)	29 (31%)	63 (66%)	0 (0%)
Did you get the help you needed in the operating room?	0 (0%)	0 (0%)	3 (3%)	11 (12%)	79 (83%)	2 (2%)
Did you feel welcome on the maternity ward?	0 (0%)	2 (2%)	2 (2%)	10 (11%)	78 (82%)	3 (3%)

The questionnaire consisted of questions about information provided by team members, team-dynamics (labor ward / operating room), participation in the delivery, leadership, and care on the maternity ward (Figs 2 and 3). Initially, the questionnaire for partners consisted of 11 questions. Analyses of the questionnaire indicated substantial misfit for the questions: “*Did the professionals engage the partner during the vaginal delivery” (question 3)* and “*Did you feel the team-dynamic was controlled during the emergency” (question 5)*. For the remaining nine questions, the fit of the Rasch model was satisfactory (Andersen z = 19.0 df = 25, p = 0.7994) and the item selection is illustrated in Fig 3.

After adjustment for multiple testing, the questions 10 and 11 showed borderline significant misfit (S2 Appendix) that was also evident in the graphical analyses of item fit (S2 Appendix). The resulting sum score (with possible values ranging from zero to 60) had a mean of 52.3 and the median was 55 (IQR: 49–58). The reliability of the scale was satisfactory (Cronbach’s alpha = 0.68).

### Step 3: Clinical factors associated with patient-perceived quality of care

The distribution of questionnaire scores for mothers and their partners with respect to the urgency classification and indication of CS is shown in Tables 5 and 6.

**Table 5:** Association between urgency classification of caesarean section and perceived quality of care measured as median questionnaire scores by mothers and partners Legend: The results represent sum-score values of perception of care reported in the questionnaires ranging from zero to 60. Pctl: Percentiles.

Emergency grade of Caesarean Section				
**Mothers**				
**Urgency classification**	**N**	**Median**	**25th Pctl**	**75th Pctl**
**1**	11	40	38	46
**2**	35	55	48	58
**3**	59	56	52	59
**Partners**				
**Urgency classification**	**N**	**Median**	**25th Pctl**	**75th Pctl**
**1**	39	35	33	36
**2**	25	31	26	34
**3**	13	32	31	34

**Table 6:** Association between indication of caesarean section and perceived quality of care measured as median questionnaire scores by mothers and partners Legend: The results represent sum-score values of perception of care reported in the questionnaires ranging from zero to 60. Pctl: Percentiles.

Indication for caesarean section				
**Mothers**				
**Indication**	**N**	**Median**	**25th Pctl**	**75th Pctl**
Dystocia of labour	**46**	**56**	**52**	**58**
Suspicion of asphyxia	**39**	**51**	**44**	**56**
Breech position	**16**	**56**	**52**	**58**
**Partners**				
**Indication**	**N**	**Median**	**25th Pctl**	**75th Pctl**
Dystocia of labour	**39**	**35**	**33**	**36**
Suspicion of asphyxia	**25**	**31**	**26**	**34**
Breech position	**13**	**32**	**31**	**34**

Mothers and their partners were more satisfied with ECS when associated with the lowest urgency. The Spearman rank correlation between the total questionnaire scores and urgency classification (1,2,3) was 0.34 (p = 0.0004) for mothers and 0.32 (p = 0.0037) for partners. Partial correlations controlling for the effect of indication for CS were 0.39 (p < 0.0001) and 0.33 (p = 0.0041) for mothers and partners, respectively.

The (total) scores differed significantly across CS urgency classifications for mothers (Kruskal-Wallis chi-square 6.8, df = 2, p = 0.0328) and for partners (Kruskal-Wallis chi-square 14.5, df = 2, p = 0.0007). Perception of care demonstrated lowest scores for most emergent CS cases (classification 1).

After adjustment for urgency classification, the quality scores differed significantly across the three indication groups for both mothers (p = 0.0006) and their partners (p < 0.0001).

For mothers, pairwise comparisons of the three indication groups showed significant differences between”Dystocia of labor” and”Suspicion of asphyxia” only after adjustment for multiple testing (p<0.05). For partners, all pairwise comparisons remained significant after adjustment for multiple testing.

## Discussion

Following ECS, mothers and their partners suggested that team-dynamics, professionalism, information provided by team-members, leadership and mother-child continuity of care were important for their perception of quality of care. A survey based on these findings demonstrated a relationship between perception of quality of care and clinical aspects of the CS. Lower urgency procedures were associated with perceptions of high-quality care; whereas procedures performed in high acuity situations, such as suspicion of fetal distress, were perceived as lower quality care.

In our study, the interviews involved a shared experience of mothers and partners, as opposed to prior studies which have focused exclusively on individual perceptions of care [4,12]. In a Scandinavian context, where the partner is present during childbirth, it is important to focus on perceptions of *both* the mother *and* her partner. Particularly in emergency deliveries, partners may feel anxious and helpless [25] and partners’ experiences may influence the mother’s overall perceptions of the birth experience. By engaging both parents, it was possible to gain insight into the shared perception of care. This methodologic choice may have introduced potential bias, since we did not distinguish the individual perspectives of mother and partner during the interview analyses and potential domination by one part is possible. However, in the weeks after delivery, the mother and her partner will most likely discuss the experience and exchange their thoughts about the emergency cesarean section, such that the shared experience may impact decision about the next pregnancy and eventually the mode of delivery.

Professionalism, as conceptualized in the interviews, may have a different meaning in the context of an emergency than in other clinical situations. In our context, professionalism related to the perception of team communication and efficiency in task completion. In other contexts, the association between professionalism and perceived quality of care relates to patient involvement and patient empowerment [32]. Hence, the components of professionalism may have varying priority depending on the clinical situation.

To our knowledge, no previous studies have explored the association between urgency classification and perceived quality of care. The time pressure of an ECS may limit the caregivers’ ability to provide the desired quality of care. However, awareness of the association between urgency and patient perceived quality of care may lead to increased emphasis on the provision of information to both mother and partner for high-urgency CS.

Our survey results demonstrated that positive perceptions of care quality were most common when labor dystocia was the indication for ECS. Consequently, perceived quality of care was lower if the CS was performed due to suspected fetal distress. This pattern may be a result of a focus on the emergency cesarean procedure and not the entire birth process. Prolonged labor has elsewhere been associated with low satisfaction when delivery is performed by ECS [20]. The higher perceived quality of care for women, who had labor dystocia, may reflect positive responses stemming from the end of a long labor without progression. On the other hand, women who expected a vaginal delivery and suddenly experienced transition to ECS because of a breech presentation, may not have experienced the exhaustion and fatigue associated with labor dystocia and, in turn, this may explain differences in observed quality of care scores, despite both conditions being of low-grade urgency.

The strengths of this study are the use of multiple sources of evidence of perceived quality of care, the combination of qualitative approaches/techniques and rigorous statistical validation, and finally the extrapolation and cross-validation with other variables, such as urgency classification and CS indications. There are also some limitations. Patient experiences may be highly context dependent. Our study included Danish mothers and their partners and similar studies in other populations are needed to provide additional information that may help identify areas that can be modified through team training and organization. Finally, the relationship between patients’ perception of care and quality of care is controversial because patient-perceived quality of care does not always reflect clinical quality of care [49]. Moreover, interventions, which improve clinical care, may not improve patient-perceived quality of care and vice versa. This notion is important, not because one perspective on quality of care should be valued higher than the other, but because they provide complimentary views on care quality that may be equally important for patients.

## Conclusion

This study identified team-dynamics, professionalism, information, safety, leadership and mother-child continuity of care, as important factors affecting perceived of care quality. These perceptions were highly influenced by CS indications and urgency. Hence, future clinical interventions to enhance patient-perceived quality of care should focus on the highest urgency CSs and emphasize the importance of team behavior.

## Supporting information

S1 Appendix Interview guide. (DOCX)

S2 Appendix Statistical analyses. (DOCX)

## Abbreviations

CS: Cesarean Section
DIF: Differential item functioning
FDR: False discovery rate
Item: Specific question in the questionnaires
ECS: Emergency Cesarean Section

## References

[1] Stadlmayr W, Schneider H, Amsler F, Burgin D, Bitzer J. How do obstetric variables influence the dimensions of the birth experience as assessed by salmon's item list [SIL-ger]? *Eur J Obstet Gynecol Reprod Biol*. 2004;115[1]:43–50. 10.1016/j.ejogrb.2003.12.015 15223164

[2] Smarandache A, Tamim H, Bohr Y, Kim T. Predictors of a negative labour and birth experience based on a national survey of canadian women. *J Womens Health*. 2017;26[4]:A50–A51. 10.1186/s12884-016-0903-2 PMC4870779 27193995

[3] Donate-Manzanares M, Rodriguez-Cano T, Gomez-Salgado J, Rodriquez-Almagro J, Hernandez-Martinez A, Barrilero-Fernandez E et al Quality of childbirth care in women undergoing labour: Satisfaction with care received and how it changes over time. *J Clin Med*. 2019;8[4]: 10.3390/jcm8040434 30934940 PMC6518019

[4] Robinson P, Salmon P, Yentis S. Maternal satisfaction. *Int J Obstet Anesth*. 1998;7[1]:32–37. 10.1016/s0959-289x(98)80026-5 15321244

[5] Hulton L, Matthews Z, Stone R. *A framework for the evaluation of quality of care in maternity services*. First ed University of Southhampton; 2000.

[6] Jea Hanefeld. Understanding and measuring quality of care: Dealing with complexity. 2017;95:368–374. 10.2471/BLT.16.179309 PMC5418826 28479638

[7] National Clinical Guideline Centre [UK]. 2012.

[8] Institute of Medicine [US] Committee on Quality of Health Care in America. 2001.

[9] Bleich S. How does satisfaction with the health-care system relate to patient experience? 2009;87:271–278. 10.2471/BLT.07.050401 PMC2672587 19551235

[10] Escuriet. Assessing the performance of maternity care in Europe: A critical exploration of tools and indicators. *BMC Health Serv Res*. 2015;15:491-015-1151-2. 10.1186/s12913-015-1151-2 PMC4631101 26525577

[11] Hodnett E. Pain and women's satisfaction with the experience of childbirth: A systematic review. *Obstet Gynecol*. 2002;186[5]:S160–S172. 10.1067/mob.2002.121141 12011880

[12] Salmon P, Drew N. Multidimensional assessment of womens experience of childbirth—relationship to obstetric procedure, antenatal preparation and obstetric history. *J Psychosom Res*. 1992;36[4]:317–327. 10.1016/0022-3999(92)90068-d 1593507

[13] Acikel A, Ozturk T, Goker A, Hayran GG, Keles GT. Comparison of patient satisfaction between general and spinal anaesthesia in emergency caesarean deliveries. *Turk J Anaesthesiol Reanim*. 2017;45[1]:41–46. 28377839 10.5152/TJAR.2017.38159 PMC5367724

[14] Stal KB, Pallangyo P, van Elteren M, van den Akker T, van Roosmalen J, Nyamtema A. Women's perceptions of the quality of emergency obstetric care in a referral hospital in rural Tanzania. *Trop Med Int Health*. 2015;20[7]:934–940. 10.1111/tmi.12496 25726853

[15] Blomquist JL, Quiroz LH, Macmillan D, McCullough A, Handa VL. Mothers' satisfaction with planned vaginal and planned cesarean birth. *Am J Perinatol*. 2011;28[5]:383–388. 10.1055/s-0031-1274508 21380993 PMC3086342

[16] Mørch Nielsen M. Perceptions of postnatal care after emergency caesarean sections. 2019;14[e-76793]:1–22.

[17] Handelzalts JE, Waldman Peyser A, Krissi H, Levy S, Wiznitzer A, Peled Y. Indications for emergency intervention, mode of delivery, and the childbirth experience. *PLoS One*. 2017;12[1]:e0169132 10.1371/journal.pone.0169132 28046019 PMC5207782

[18] Lucas A. Information for women after CS: Are they getting enough? *RCM Midwives*. 2004;7[11]:472–475. 15612178

[19] Campillo-Artero C, Serra-Burriel M, Calvo-Perez A. Predictive modeling of emergency cesarean delivery. *PLoS One*. 2018;13[1]:e0191248 10.1371/journal.pone.0191248 29360875 PMC5779661

[20] Bossano CM, Townsend KM, Walton AC, Blomquist JL, Handa VL. The maternal childbirth experience more than a decade after delivery. *Am J Obstet Gynecol*. 2017. 10.1016/j.ajog.2017.04.027 28455080

[21] Carquillat P, Boulvain M, Guittier MJ. How does delivery method influence factors that contribute to women's childbirth experiences? *Midwifery*. 2016;43:21–28. 10.1016/j.midw.2016.10.002 27825057

[22] Fisher C, Hauck Y, Fenwick J. How social context impacts on women's fears of childbirth: A western Australian example. *Soc Sci Med*. 2006;63[1]:64–75. 10.1016/j.socscimed.2005.11.065 16476516

[23] National Perinatal epidemiology unit. Audit commission, UK. first class deliveries. A national survey of women's views of maternity care. http://webarchive.nationalarchives.gov.uk/20150410163038/http://archive.audit-commission.gov.uk/auditcommission/aboutus/publications/pages/national-reports-and-studies-archive.aspx.html.

[24] O'Cathain A, Knowles E, Nicholl J. Measuring patients' experiences and views of the emergency and urgent care system: Psychometric testing of the urgent care system questionnaire. *BMJ Qual Saf*. 2011;20[2]:134–140. 10.1136/bmjqs.2009.036574 21209128

[25] Rosich-Medina A. Paternal experiences of pregnancy and labour. 2007;15[2]:66–74.

[26] Gawlik S, Müller M, Hoffmann L, Dienes A, Reck C. Assessing birth experience in fathers as an important aspect of clinical obstetrics: How applicable is Salmon׳s item list for men? *Midwifery*. 2015;31[1]:221–228. 10.1016/j.midw.2014.08.013 25242108

[27] Salmon P, Miller R, Drew N. Womens anticipation and experience of childbirth—the independence of fulfilment, unpleasantness and pain. *Br J Med Psychol*. 1990;63:255–259. 10.1111/j.2044-8341.1990.tb01617.x 2245201

[28] Soriano-Vidal FJ, Oliver-Roig A, Cabrero-Garcia J, Congost-Maestre N, Dencker A, Richart-Martinez M. The spanish version of the childbirth experience questionnaire (CEQ-E): Reliability and validity assessment. *BMC Pregnancy Childbirth*. 2016;16[1]:372 10.1186/s12884-016-1100-z 27884123 PMC5123212

[29] Spaich S, Welzel G, Berlit S, Temerinac D, Tuschy B, Kehl S. Mode of delivery and its influence on women's satisfaction with childbirth. *European Journal of Obstetrics & Gynecology and Reproductive Biology*. 2013;170[2]:401–406. 23962715 10.1016/j.ejogrb.2013.07.040

[30] Triolo PK, Hansen P, Kazzaz Y, Chung H, Dobbs S. Improving patient satisfaction through multidisciplinary performance improvement teams. *J Nurs Adm*. 2002;32[9]:448–454. 10.1097/00005110-200209000-00006 12360116

[31] Waldenstrom U. Experience of labor and birth in 1111 women. *J Psychosom Res*. 1999;47[5]:471–482. 10.1016/s0022-3999(99)00043-4 10624845

[32] Wiggins MN, Coker K, Hicks EK. Patient perceptions of professionalism: Implications for residency education. *Med Educ*. 2009;43[1]:28–33. 10.1111/j.1365-2923.2008.03176.x 19148978

[33] Gehlbach H, Artino AR Jr, Durning S. AM last page: Survey development guidance for medical education researchers. *Acad Med*. 2010;85[5]:925 10.1097/ACM.0b013e3181dd3e88 20520050

[34] Braun V, Clarke V. Using thematic analysis in psychology. 2006;3:77–101.

[35] Lundsgaard KS, Tolsgaard MG, Mortensen OS, Mylopoulos M, Ostergaard D. Embracing multiple stakeholder perspectives in defining trainee competence. *Acad Med*. 2019;94[6]:838–846. 10.1097/ACM.0000000000002642 30730374

[36] Malterud K. Systematic text condensation: A strategy for qualitative analysis. *Scand J Public Health*. 2012;40[8]:795–805. 10.1177/1403494812465030 23221918

[37] Hsieh HF, Shannon SE. Three approaches to qualitative content analysis. *Qual Health Res*. 2005;15[9]:1277–1288. 10.1177/1049732305276687 16204405

[38] Cleland J S AD. *Researching medical education*. UK: The association for the Study of Medical Education; 2015.

[39] RCOG. Classification of urgency of caesarean section—a continuum of risk [good practice no. [11]2010;11.

[40] Van der linden WJ, Hambleton RK. *Handbook of modern item response theory*. Springer, New York, NY; 1997 10.1007/978-1-4757-2691-6.

[41] Christensen KB, Kreiner S, Mesbah M. *Rasch models in health*. First ed Wiley; 2013.

[42] Andersen E. Goodness of fit test for Rasch model. *Psychometrika*. 1973;38[1]:123–140.

[43] Kreiner S. A note on item-restscore association in rasch models. *Appl Psychol Meas*. 2011;35[7]:557–561.

[44] Christensen KB, Makransky G, Horton M. Critical values for yen's Q(3): Identification of local dependence in the rasch model using residual correlations. *Appl Psychol Meas*. 2017;41[3]:178–194. 10.1177/0146621616677520 29881087 PMC5978551

[45] Holland P, Wainer H. *Differential item functioning*. Hillsdale, New Jersey: Lawrence Erlbaum associates; 1993.

[46] Kelderman H. Loglinear rasch model tests. *Psychometrika*. 1984;49[2]:223–245.

[47] Kolen MJ, Brennan RL. *Test equating*, *scaling*, *and linking*: *Methods and practices*. Third ed USA: Springer New York; 2014 https://www-scopus-com.ep.fjernadgang.kb.dk/record/display.uri?src=s&origin=cto&ctoId=CTODS_1043371273&stateKey=CTOF_1043371314&eid=2-s2.0-85028499171.

[48] Benjamini Y, Hochberg Y. Controlling the false discovery rate—a practical and powerful approach to multiple testing. *J R Stat Soc Ser B-Methodol*. 1995;57[1]:289–300.

[49] Tsai TC, Orav EJ, Jha AK. Patient satisfaction and quality of surgical care in US hospitals. *Ann Surg*. 2015;261[1]:2–8. 10.1097/SLA.0000000000000765 24887985 PMC4248016

