# Supplementary material for: Making the best of the worst: Care quality during emergency cesarean sections
Source: PLoS One. 2020 Feb 21;15(2):e0227988. doi: 10.1371/journal.pone.0227988 (PMC7034794; doi:10.1371/journal.pone.0227988)
Supplement: S1 Appendix — (DOCX) [file pone.0227988.s001.docx]

## Appendix 1: Interview guide

- How did you experience an emergency cesarean section?
- How did you experience the communication within the team of professionals?
- How did you experience the information you received?
- How would you describe the atmosphere?
- Did you feel safe?
- Did you feel frightened?
- Did you sense a clear leadership during the emergency cesarean section?
- How would you describe the contact with your child?
- How would you describe the importance of talking to the doctor who performed the CS?
- Can you think of anything that you would recommend us to do differently in the future?
