# Supplementary material for: Making the best of the worst: Care quality during emergency cesarean sections
Source: PLoS One. 2020 Feb 21;15(2):e0227988. doi: 10.1371/journal.pone.0227988 (PMC7034794; doi:10.1371/journal.pone.0227988)

## Appendix 2: Statistical analyses

#### **Appendix A:** Details of the of Rasch analysis of the scale for **mothers**

#### Initial analyses of the 17 items for the mothers revealed misfit for two items ‘controlled atmosphere on the labour ward’ and ‘controlled atmosphere in operating room’ (Figure A1).

#### **Figure A1**. Graphical evaluation of item fit. Observed item means plotted against grouped total scale scores for two mis-fitting items (women).


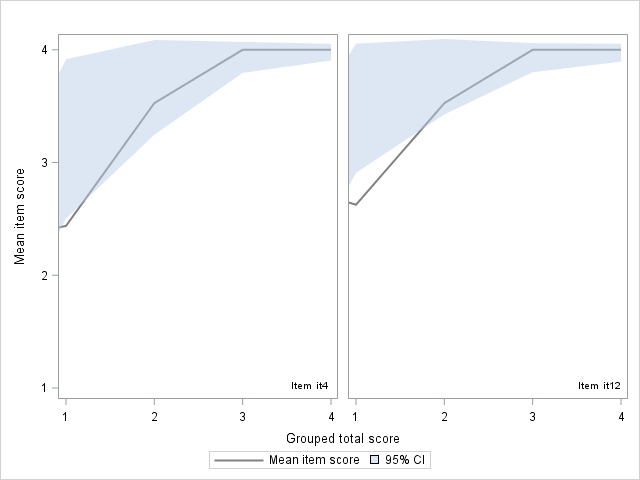


When these two items (item 4 and item 12) were excluded, the remaining 15 items formed a scale with satisfying fit to the Rasch model (Andersen *z*= 60.1, df=49, P=0.1329).
Individual item fit was acceptable; with no significant item fit statistics after control for multiple testing (Table A1) and only minor evidence of misfit (Figure A2).

#### **Table A1**. Item fit statistics and test of differential item functioning for the 15 items in the final scale (mothers).

| **Item** | | Item Fit | | | DIF | | | | | |
| --- | --- | --- | --- | --- | --- | --- | --- | --- | --- | --- |
|  |  |  |  |  | Indication | | | Emergency grade | | |
|  |  | **Obs** | **exp** | **FDR** | **clr** | **df** | **FDR** | **clr** | **df** | **FDR** |
| 1 | Information on the labour ward | 0.40 | 0.46 | 0.6710 | 1.3 | 4 | 0.9150 | 6.0 | 4 | 0.3557 |
| 2 | Took care of my needs on the labour ward | 0.58 | 0.47 | 0.6710 | 2.2 | 2 | 0.7099 | 2.9 | 6 | 0.8230 |
| 3 | Atmosphere, Labour ward, - calm | 0.74 | 0.63 | 0.4510 | 21.8 | 8 | 0.0795 | 14.2 | 8 | 0.2090 |
| 4 | Atmosphere, Labour ward, - controlled |  |  |  |  |  |  |  |  |  |
| 5 | Atmosphere, Labour ward, - hectic | 0.79 | 0.65 | 0.3646 | 8.4 | 8 | 0.7412 | 13.9 | 8 | 0.2090 |
| 6 | Atmosphere, Labour ward, - nervous | 0.82 | 0.61 | 0.4510 | 11.5 | 8 | 0.5184 | 12.4 | 8 | 0.2891 |
| 7 | Atmosphere, Labour ward, - chaotic | 0.72 | 0.63 | 0.6710 | 12.1 | 6 | 0.2209 | 9.7 | 8 | 0.3944 |
| 8 | I felt safe | 0.46 | 0.51 | 0.6710 | 5.7 | 8 | 0.8460 | 8.8 | 8 | 0.4011 |
| 9 | Contact to midwife | 0.47 | 0.49 | 0.8431 | 0.3 | 4 | 0.9913 | 4.6 | 4 | 0.4011 |
| 10 | Clear leadership | 0.37 | 0.53 | 0.3322 | 16.3 | 8 | 0.1945 | 22.6 | 8 | **0.0300** |
| 11 | Atmosphere Operating room,  - calm | 0.80 | 0.59 | 0.1775 | 5.8 | 8 | 0.8460 | 8.6 | 8 | 0.4011 |
| 12 | Atmosphere, Operating room  - controlled |  |  |  |  |  |  |  |  |  |
| 13 | Atmosphere, Operating room  - hectic | 0.60 | 0.60 | 0.9200 | 3.0 | 6 | 0.9150 | 16.3 | 6 | 0.0620 |
| 14 | Atmosphere, Operating room  - nervous | 0.74 | 0.57 | 0.4510 | 10.1 | 8 | 0.6390 | 10.5 | 8 | 0.3557 |
| 15 | Atmosphere, Operating room - chaotic | 0.65 | 0.61 | 0.8431 | 5.0 | 6 | 0.8207 | 10.4 | 8 | 0.3557 |
| 16 | Felt welcomed on maternity ward | 0.33 | 0.47 | 0.4510 | 12.8 | 4 | 0.0938 | 15.8 | 4 | **0.0300** |
| 17 | Had all needed help on maternity ward | 0.31 | 0.46 | 0.4510 | 1.4 | 2 | 0.8207 | 10.4 | 4 | 0.1275 |

DIF: Differential item functioning; FDR: False discovery rate; Item: Specific question in the questionnaires

#### **Figure A2**. Graphical evaluation of item fit for the final 15 item scale (mothers). Observed item means plotted against grouped total scale score.


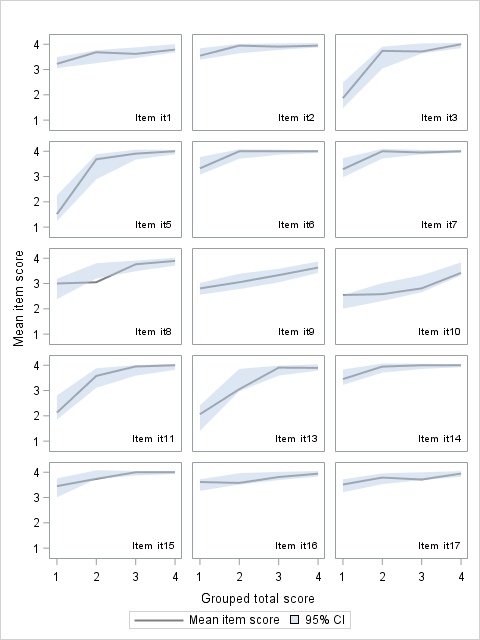


There was evidence of local response dependence for three item pairs ‘calm atmosphere on labour ward’ and ‘hectic atmosphere on labour ward’; ‘calm atmosphere in operating room’ and ‘hectic atmosphere in operating room’; ‘Did nurses welcome you on the maternity ward’ and ‘Did you get all the help you needed on the maternity ward’ (results not shown).

The distribution of the total score in the final scale is shown in Figure A3.

#### **Figure A3**. Histogram of total score of the perceived quality of care scale (mothers). Maximum value of scale score is 60.


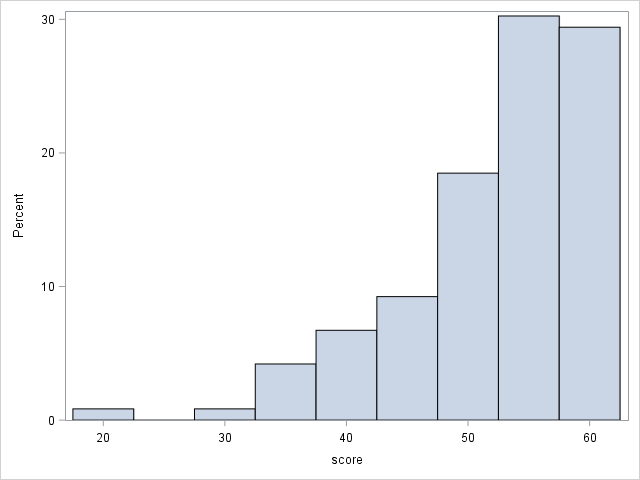


#### **Figure A4**. Equated scores for mothers. Item 10 and item 16 inflate the total score in the group “grade of CS”.


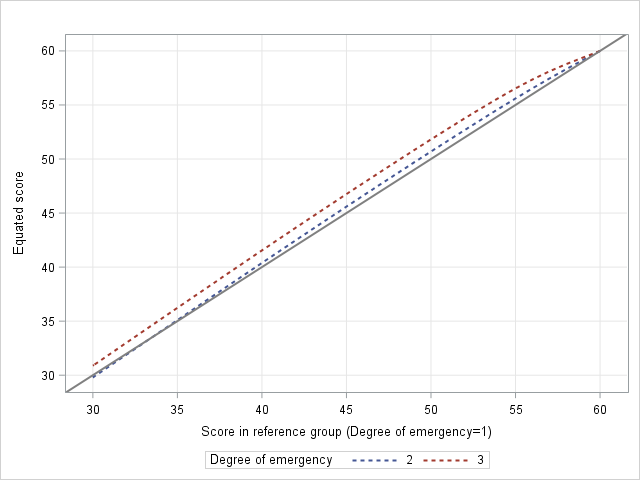


#### **Appendix B**: Details of the of Rasch analysis of the scale for **partners**

#### Initial analyses of the 11 items for the partners revealed misfit for two items 'did midwife include you' and 'controlled atmosphere in labour ward' (Figure B1).

#### **Figure B1**. Graphical evaluation of item fit. Observed item means plotted against grouped total scale score for two mis-fitting items (partners).


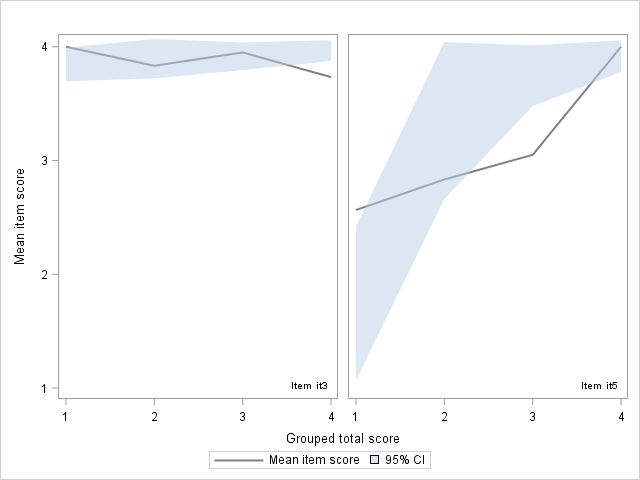


When these two items were excluded the remaining nine items formed a scale with satisfying fit to the Rasch model (Andersen *z*=19.0, df=25, P=0.7994).

Individual item fit was acceptable, with no significant item fit statistics after control for multiple testing (Table B1), but some evidence of misfit in the graphical analysis of item fit (Figure B2).

#### **Table B1**. Item fit statistics and tests of differential item functioning for the nine items in the final scale (partners).

| **Item** | | Item Fit | | | DIF | | | | | |
| --- | --- | --- | --- | --- | --- | --- | --- | --- | --- | --- |
|  |  |  |  |  | Indication | | | Emergency grade | | |
|  |  | **Obs** | **exp** | **FDR** | **clr** | **f** | **FDR** | **clr** | **f** | **FDR** |
| 1 | Information on the labour ward | 0.55 | 0.56 | 0.8960 | 1.1 | 4 | 0.9709 | 4.2 | 4 | 0.6874 |
| 2 | Took care of my needs on the labour ward | 0.54 | 0.52 | 0.8960 | 2.2 | 2 | 0.6124 | 2.2 | 6 | 0.9042 |
| 3 | The professionals involved the partner |  |  |  |  |  |  |  |  |  |
| 4 | Atmosphere, Labour ward,  - calm | 0.70 | 0.63 | 0.6666 | 18.4 | 8 | 0.1665 | 7 | 8 | 0.7974 |
| 5 | Atmosphere, Labour ward,  - controlled |  |  |  |  |  |  |  |  |  |
| 6 | Atmosphere, Labour ward,  - hectic | 0.71 | 0.64 | 0.6666 | 11 | 8 | 0.4518 | 11.4 | 8 | 0.5325 |
| 7 | Atmosphere, Labour ward,  - nervous | 0.65 | 0.56 | 0.7061 | 10.8 | 6 | 0.2820 | 9.5 | 8 | 0.6728 |
| 8 | Atmosphere, theatre,  - chaotic | 0.68 | 0.53 | 0.6666 | 5.6 | 8 | 0.8878 | 4.6 | 8 | 0.9006 |
| 9 | Felt safe | 0.60 | 0.54 | 0.7061 | 0.5 | 4 | 0.9709 | 1.7 | 4 | 0.9006 |
| 10 | Felt supported during the emergency. Operating theatre | 0.17 | 0.50 | 0.0585 | 14.3 | 8 | 0.2820 | 21.6 | 8 | 0.0513 |
| 11 | Felt welcomed at maternity ward | 0.22 | 0.53 | 0.0585 | 7.5 | 8 | 0.7307 | 18.1 | 8 | 0.0932 |

DIF: Differential item functioning; FDR: False discovery rate; Item: Specific question in the questionnaires

#### **Figure 2**. Graphical evaluation of item fit for the final nine item scale (partners). Observed item means plotted against grouped total scale score.


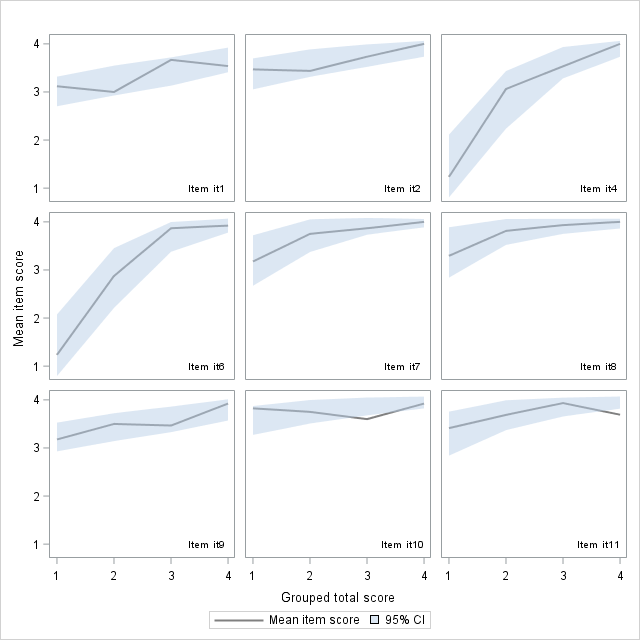


No evidence of local response dependence or of differential item functioning was found.

The distribution of the total score in the final scale is shown in Figure 3.

#### **Figure B3**. Histogram showing the distribution of the total score of perceived quality of care for partners. Maximum value of scale score is 36.


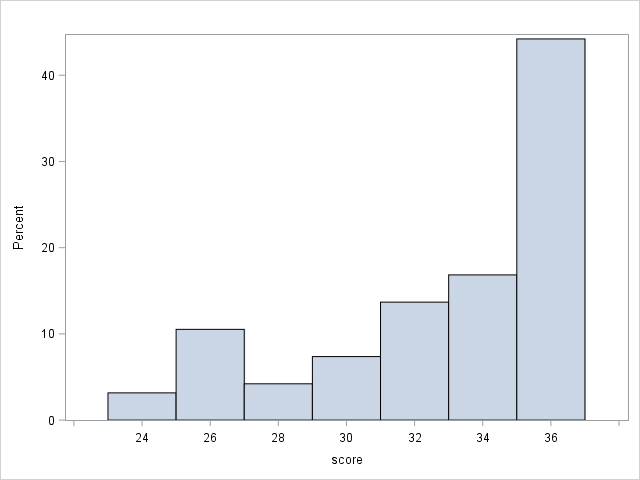

Supplement: S2 Appendix — (DOCX) [file pone.0227988.s002.docx]
